# MicroRNA Regulation of the Synaptic Plasticity-Related Gene *Arc*

**DOI:** 10.1371/journal.pone.0041688

**Published:** 2012-07-26

**Authors:** Karin Wibrand, Balagopal Pai, Taweeporn Siripornmongcolchai, Margarethe Bittins, Birgitte Berentsen, May Lillian Ofte, Arwed Weigel, Kai Ove Skaftnesmo, Clive R. Bramham

**Affiliations:** Department of Biomedicine and K.G. Jebsen Centre for Research on Neuropsychiatric Disorders, University of Bergen, Bergen, Norway; University of Barcelona, Spain

## Abstract

Expression of activity-regulated cytoskeleton associated protein (Arc) is crucial for diverse types of experience-dependent synaptic plasticity and long-term memory in mammals. However, the mechanisms governing Arc-specific translation are little understood. Here, we asked whether Arc translation is regulated by microRNAs. Bioinformatic analysis predicted numerous candidate miRNA binding sites within the Arc 3′-untranslated region (UTR). Transfection of the corresponding microRNAs in human embryonic kidney cells inhibited expression of an Arc 3′UTR luciferase reporter from between 10 to 70% across 16 microRNAs tested. Point mutation and deletion of the microRNA-binding seed-region for miR-34a, miR-326, and miR-19a partially or fully rescued reporter expression. In addition, expression of specific microRNA pairs synergistically modulated Arc reporter expression. In primary rat hippocampal neuronal cultures, ectopic expression of miR-34a, miR-193a, or miR-326, downregulated endogenous Arc protein expression in response to BDNF treatment. Conversely, treatment of neurons with cell-penetrating, peptide nucleic acid (PNA) inhibitors of miR-326 enhanced Arc mRNA expression. BDNF dramatically upregulated neuronal expression of Arc mRNA and miR-132, a known BDNF-induced miRNA, without affecting expression of Arc-targeting miRNAs. Developmentally, miR-132 was upregulated at day 10 *in vitro* whereas Arc-targeting miRNAs were downregulated. In the adult brain, LTP induction in the dentate gyrus triggered massive upregulation of Arc and upregulation of miR-132 without affecting levels of mature Arc-targeting miRNAs. Turning to examine miRNA localization, qPCR analysis of dentate gyrus synaptoneurosome and total lysates fractions demonstrated synaptic enrichment relative to small nucleolar RNA. In conclusion, we find that Arc is regulated by multiple miRNAs and modulated by specific miRNA pairs *in vitro*. Furthermore, we show that, in contrast to miR-132, steady state levels of Arc-targeting miRNAs do not change in response to activity-dependent expression of Arc in hippocampal neurons *in vitro* or during LTP *in vivo*.

## Introduction

Mounting evidence supports a role for Arc as a vertebrate-specific gene specialized for mediating activity-dependent synaptic plasticity [1]–[4]. Arc synthesis is required for different forms of protein synthesis-dependent synaptic plasticity including long-term potentiation (LTP), long-term depression (LTD), and homeostatic plasticity, as well as postnatal development of the visual cortex and multiple forms of long-term memory [5]–[9]. Furthermore, animal disease models link dysregulation of Arc protein expression to Angelman mental retardation syndrome, Alzheimer's disease, syndromic autism, seizure development, and anxiety-like behavior [10]–[14].

Arc is rapidly induced as an immediate early gene whereupon a fraction of the new RNA is transported to dendrites for local storage, translation, or decay, probably in a stimulus-specific and context-specific manner [3], [7], [15]–[18]. Each step in the life of Arc mRNA is subject to tight control, but the mechanisms are still only partly understood. Recently, microRNAs have emerged as major modulators of protein expression levels in the mammalian brain. The present study sought to elucidate a possible role for microRNAs as modulators of Arc.

miRNAs are small endogenous non-coding regulatory RNAs (21–23 nt) that bind to imperfectly complementary sequences in the 3′UTR of target genes and inhibit protein expression. miRNAs function by recruiting the RNA-induced silencing complex (miRISC), which represses translation or promotes mRNA decay [19], [20]. Many new brain-specific miRNA families have appeared in the vertebrate evolutionary line, with diversity increasing in non-human primates and humans, suggesting that miRNA evolution is an ongoing process linked to greater brain complexity [21], [22]. The vertebrate genome harbors more than 1500 miRNA genes and each miRNA is bioinformatically predicted to target hundreds of mRNAs. This suggests an elaborate net of regulation with many mRNAs targeted by multiple microRNAs, but so far relatively few miRNA/target interactions have been experimentally validated in the nervous system. MicroRNA activity can be modulated by changes in mature miRNA biogenesis, access of miRNA to target, or regulation of RISC effector proteins [23]–[28]. In recent years, roles for specific microRNAs in neurogenesis, dendritic spine morphogenesis, synaptic regulation, plasticity, and memory have been demonstrated [29]–[38].

As a recently evolved “master” regulator of synaptic plasticity, Arc is an excellent candidate for elucidating microRNA regulation and function in the context of complex behaviors and cognition. The present study demonstrates miRNA regulation of Arc and suggests a graded control mediated by multiple miRNAs with different developmental and subcellular expression profiles. Furthermore, activity-dependent upregulation of Arc is not associated with altered miRNA expression *in vitro* or *in vivo*.

## Materials and Methods

### Ethics statement

Animal experiments were carried out in accordance with the European Community Council Directive of 24 November 1986 (86/609/EEC) and approved by the Norwegian Committee for Animal Research.

### Cell culture

Human embryonic kidney cells (HEK293T; American Type Culture Collection number CRL-11268; www.atcc.org) were maintained in Dulbecco's modified Eagle medium (DMEM, Sigma) supplemented with 10% heat-inactivated fetal calf serum (Sigma), 100 U/ml penicillin (Sigma), 100 µg/ml streptomycin (Sigma) and 1 mM L-glutamine at 37°C and 5% CO_2_. Cells were seeded in 96-well plates for luciferase reporter assays.

Hippocampal neurons in dispersed culture were prepared with slight modifications from the protocol originally described by Banker and co-workers (Banker and Cowan 1977; Kaech and Banker 2006). Hippocampi of Wistar rat embryos (E18) were dissected and dissociated by trypsin treatment followed by trituration. After removal of trypsin, neurons were plated at a density of 60,000 cells/well on 10 mm diameter cover slips for imaging experiments or at a density of 240,000 cells/well in a 6-well plate for biochemical analysis. Both cover slips and 6-well plates were precoated with poly-D-lysine (Sigma). The cultures were maintained in MEM (M2279, Sigma) growth medium supplemented with B-27 supplement (B-27® Supplement, Invitrogen), sodium bicarbonate, glucose, and pyruvate, that had been conditioned on astrocyte cultures for 3 days. Half of the neuronal growth medium was replaced with fresh growth medium twice a week. BDNF (100 ng/ml) was applied for 30 minutes, 3 hours, or 4 hours.

### Prediction of microRNAs targeting the Arc 3′UTR

A list of 16 microRNAs predicted to target the Arc 3′UTR was generated from a larger set of microRNAs obtained by several different bioinformatic tools. RNAHybrid with default settings was used in combination with Crosslink software. Candidate microRNAs were also predicted by TargetScan (Release 4.2, April 2008) and miRanda (Release September 2008).

### Cloning and plasmids

#### MicroRNA constructs

Human and rat genomic DNA was isolated using Gene Elute Genomic DNA Mini Kit (Sigma) and used as template for cloning of a panel of pri-microRNAs. The pri-miRNA was cloned into the lentiviral p232 construct kindly provided by Stephen Elledge. For overexpression of miR-19a precursor a commercial construct (RmiR6091-MR04) was used (GeneCopoeia, Rockville, MD).

#### Gaussia Luciferase reporter vector

The reporter vector pcDNAGluc encoding secreted Gaussia luciferase (Gluc) was made by subcloning a HindIII-Not1 Gluc fragment from pNEBR-X1Gluc (NEB, Ipswich, USA) into pcDNA3.1 (Invitrogen, Carlsbad USA). The complete Arc 3′UTR was amplified from rat brain cDNA using specific primers with Xho1 and EcoRI overhang and cloned into the reporter vector pcDNA3.1Gluc downstream of the Gluc reporter gene.

#### Site-directed mutation (SDM) constructs

Site-directed mutagenesis of the microRNA seed-binding regions (corresponding to three nucleotides in the seed sequence) and deletion constructs were generated using the QuickChange SDM kit (Stratagene, La Jolla, CA).

All constructs were confirmed by DNA sequencing. Primers are available on request.

### Cell transfection experiments and reporter assays

#### Reporter luciferase assay

HEK-293T cells (American Type Culture Collection number CRL-11268) were seeded in 96-well plates and transiently transfected with Gaussia luciferase reporter (20 ng) constructs, microRNA vectors (100 ng), and gWIZ SEAP Mammalian Expression Vector (10 ng) (P050200, Genlantis, San Diego, CA, USA) using FuGENE® HD transfection reagent (Roche). Gaussia luciferase is a secreted luciferase and 48 hours after transfection medium was collected and Gluc activity was measured using BioLux® Gaussia Luciferase Assay Kit (NEB, Ipswich, MA) according to the manufacturer's protocol. The activity of gWIZ Secreted Alkaline Phosphatase (SEAP) was used for normalization of transfection efficiency.

#### Transfection of primary hippocampal cell cultures with microRNA vectors

Two slightly different experiments were carried out to look at the effect of endogenous Arc expression after overexpression of microRNAs. After 7 days *in vitro* (DIV7), neurons were transfected with plasmids expressing DsRed only (empty vector), ds-Red-miR-150, DsRed-miR-326 or DsRed-miR-193a using Lipofectamine-2000 Reagent (Invitrogen). One day before transfection the medium was changed and replaced with fresh medium containing 2 mg/ml vitamin C. In the second set of experiments neurons were transfected with DsRed only, DsRed-miR150, or DsRed-miR34a using Lipofectamine LTX and Plus Reagent (Invitrogen) according to manufacturer's instructions. Three days after transfection, human BDNF (Alomone labs B-250) was added to a final concentration of 100 ng/ml for four hours to induce expression of Arc [39]. For immunocytochemistry, cells were washed with phosphate buffered saline (PBS), fixed with 4% paraformaldehyde/sucrose/PBS for 15 minutes, treated with 50 mM ammonium chloride/PBS for 10 minutes, permeabilized with 0.1% Triton X-100/PBS for 5 minutes, and blocked with 0.5% bovine serum albumin (BSA)/PBS for 30 minutes. Antibodies were diluted in 0.5% BSA/PBS. Primary antibody: Arc C-7 (Santa Cruz Biotechnology, sc-17839), 1∶200, overnight at 4°C. Secondary antibody: donkey anti-mouse coupled to Alexa Fluor 647 (Invitrogen A31571), 1∶500, 30 minutes at room temperature. In the first set of experiments Phalloidin FITC (Sigma, P5282) was added at a 1∶40 dilution to simplify the identification of the cells. Coverslips were washed in ddH_2_O and mounted in ProLongGold antifade mounting medium containing DAPI (Invitrogen).

### Imaging and image analysis

Imaging was done with a Zeiss Axio Imager Z1 upright fluorescence microscope equipped with a mercury arc lamp (HXP 120), a 40× oil immersion objective (EC Plan-NEO FLUAR 40×/1.3 Oil), single pass fluorescent filters for DAPI (488049-0000), DsRed (1114-101), infrared (488050-0000) and FITC (1114-459) spectra, and a CCD camera (AxioCam MRm). For each experiment, exposure times were carefully chosen to avoid saturation and all images were taken on the same day using the same exposure times. Microscopy images were analysed using the open source software CellProfiler (www.cellprofiler.org). For the miR-34a experiments nuclei were automatically identified from the DAPI images. They were used as seed regions in the Arc/AF647-stained image to automatically identify the outlines of the neurons using a watershed segmentation algorithm. The image was discarded when the segmentation algorithm failed. For each cell, the area, mean Arc/AF647 intensity, mean DsRed intensity and the respective standard deviations were measured. For miR-326 and miR-193a, CellProfiler threshold detection was used to separate the nuclear and cytoplasmic signals. In order for the nucleus-seed-algorithm to work for all cells, the single channels (AF647, DsRed) were smoothed, weighed, added and then the nuclei are used as a seed in this calculated image.

Data analysis: Using Microsoft Excel, the average intensity of DsRed in non-transfected cells was determined from the cumulative frequency plot, and the miR-34a transfected cells were normalized to that value. Arc data were also normalized to the average value in non-transfected cells. miR-326 and miR-193a experiments were similarly analyzed but in this series mean values of cytoplasmic pixels from the 20 highest DsRed expressing cells were compared with the 20 lowest.

### PNA microRNA inhibitors

PNA modified antisense oligonucleotides (PNA-AS) complementary to miR-326, miR-34a and miR-19a were purchased from Panagene Inc. (Daejeon, Korea). The oligonucleotide is coupled to a cell-penetrating peptide RRRQRRKKR (PNA™ miRNA inhibitors) in order to facilitate cellular uptake in difficult to transfect cells such as primary neurons. The peptide is linked to the microRNA antisense sequence by an ethylene glycol linker (EAAE linker). PNA-AS was transfected at a final concentration of 500 nM using Lipofectamine 2000. The transfection mix was replaced with conditioned growth medium after 3 hours. Cells were harvested after 48 hours for protein or RNA extraction.

### Immunoblot analysis

Antibodies used for western blot analysis were: Arc C7 (sc-17839,1∶500, Santa Cruz Biotech, Santa Cruz CA, USA) and GAPDH (sc-32233,1∶1000, Santa Cruz Biotech, Santa Cruz CA, USA). Equal amount of samples were loaded onto 10% SDS-PAGE gels and run for 2.5 to 3 hours at a constant voltage of 100 V. Separated proteins were subjected to western blotting. For western blotting, separated proteins were transferred to HyBond ECL nitrocellulose membrane (Amersham, Little Chalfont,UK) at a constant voltage of 100 V for 1.5 hours. Membranes were stained with Ponceau to check for proper transfer followed by blocking with blocking buffer (5% BSA, 0.1% Tween and Tris-buffered saline (TBST) for 1 hour on a gyro-rocker at room temperature. The primary and the secondary antibodies were diluted in 2% BSA in 0.1% TBST. The membranes were incubated with primary antibody overnight at 4°C with constant shaking. Following three washes with TBST, blots were incubated for 1 hour in horseradish peroxidase conjugated secondary antibody dissolved in 2% BSA in TBST. The blots were washed three times with TBST and once with 1× TBS and proteins visualized using enhanced chemiluminescence (ECL Western Blotting Analysis System, Amersham Pharmacia Biotech, Norway).

### Synaptoneurosome preparation

Synaptoneurosomes were prepared by the filtration method [40], [41]. Dentate gyrus tissue samples were homogenized in a dounce homogenizer in homogenization buffer (final concentration of 50 mM HEPES pH 7.4, 125 mM NaCl, Sucrose 100 mM, and EGTA 10 mM). Before homogenization one tablet of protease inhibitor (Complete, Mini; Protease Inhibitor Cocktail Tablets, Roche, Mannheim, Germany) and 10 µl RNAase inhibitor (RiboLock™ RNAse Inhibitor, Fermenta, St. Leon-Rot, Germany) was added to 10 ml of homogenization buffer. An aliquot was removed for total homogenate comparison. The remaining homogenate was centrifuged at 2000 *g* for 1 minute at 4°C. The supernatant was filtered through two layers of 100 µm nylon filters (NY1H04700, Millipore, Billerica, MA, USA). A second filtration was done through one layer of 5 µm PVDF membrane (SVLP01300, Millipore, Billerica, MA, USA). The filtrate was then centrifuged at 1000 *g* for 10 minutes at 4°C. The pellet containing the synaptoneurosome fraction was dissolved in homogenization buffer and used for protein analysis or RNA extraction.

### In vivo electrophysiology

The electrophysiology procedures have been detailed elsewhere [42], [43]. Adult male Sprague-Dawley rats weighing 250 g −350 g were anesthetized with urethane (1.5 g/kg i.p.) and electrodes were inserted for selective stimulation of the medial perforant path and recording of evoked potentials in the hilar region of dentate gyrus. The HFS paradigm for LTP induction consisted of eight pulses at 400 Hz, repeated four times at 10 s intervals. Three sessions of HFS were given, with 5 minutes between each HFS. LTP induction was performed for various intervals of time before dissecting out the dentate gyri. Signals from the dentate hilus were amplified, filtered (1 Hz to 10 kHz), and digitized (25 kHz). Acquisition and analysis of field potentials were accomplished using Data Wave Technologies Work Bench Software (Longmont, CO). The maximum slope of the fEPSP and the amplitude of the population spike measured from its negative going apex to the tangent line, joining the first two positive peaks were measured and the averages of four consecutive responses were obtained. Analysis of variance (ANOVA) for repeated measures followed by a post hoc Scheffé test was used for statistical analysis of group effects. Statistics were based on values obtained during the 5 minutes at the end of baseline.

### RNA extraction, reverse transcription and real-time PCR

#### RNA extraction

Total RNA was extracted from primary hippocampal cell cultures with TRIzol®reagent (Life technologies, Invitrogen, Carlsbad, CA, USA) according to the manufacturer's instructions. RNA was dissolved in 1 mM EDTA and treated with TURBO DNA-free™ Kit (Ambion, Austin, TX) prior to RT-PCR. To validate the inhibition of microRNAs after PNA AS transfection, RNA enriched in small RNAs was purified using PureLink® miRNA Isolation Kit (Life technologies, Invitrogen, Carlsbad, CA, USA).

#### Quantitative real-time PCR for *Arc*

For measurements of Arc mRNA, 150 ng of total RNA was used as input for cDNA synthesis using SuperScript® III First Strand Synthesis System (Life technologies, Invitrogen, Carlsbad, CA, USA). The cDNA was diluted 10-fold and 3 µl was used as template in semi-quantitative real-time PCR together with Lightcycler®480 DNA SYBR Green Master Mix (Roche). Semiquantitative real-time PCR was performed on a Roche Lightcycler®480. Samples were assayed in triplicates and normalized to the geometric mean of polyubiquitin and hypoxanthine phosphoribosyltransferase (HRPT) or the geometric mean of polyubiqutin, HRPT and cyclophilin [44]. Primer sequences are found in Table S1.

#### Taqman miRNA real-time qPCR

Changes in mature microRNA levels were determined using the TaqMan® MicroRNA Reverse Transcription Kit and TaqMan® microRNA Assays (Applied Biosystems, Foster City, CA) according to manufacturer's protocol. 15 µl of cDNA was generated from 30 ng of total RNA and 3 µl of a 15-fold dilution was used for real-time PCR reactions. Y1 and snoRNA has been used for normalization.

Changes in relative concentration was calculated with the second derivative maximum method 2^−ΔCT^. ΔCT was calculated by subtracting the CT of the housekeeping gene from the CT of the gene of interest. Fold change was generated using the equation 2^−ΔΔCT^. Student's *t*-test was used for the statistical analyses.

### microRNA *in situ* hybridization

Rats were intracardially perfused with 4% paraformaldehyde (PFA). The brain was removed and submerged sequentially in 4% PFA for 24 hours at 4°C and 30% sucrose for 48 hours at 4°C. On the following day the brains were frozen in CO_2_ gas and 30 µm-thick coronal sections were cut on a Leica CM3050S (cryo-microtome) using Richard-Allan Sec5e blades. Sections were immediately stored in phosphate buffer containing 0.1% azide at 4°C. *In situ* hybridization was performed on 30 µm-thick floating sections using locked nucleic acid (LNA) probes as described previously [23], [45]. The LNA probes were a gift from Dr Thomas Tuschl's lab (Rockefeller University, NY, USA).

Sections were rinsed in TBS (Tris-buffered saline) and incubated with proteinase K for 5 minutes at 37°C, washed twice in TBS, then post-fixed for 5 minutes in 4% PFA. After washing once in 0.2% Glycine/TBS and twice in TBS, sections were incubated in freshly prepared 1-methylimidazole solution, and then immersed in 1–ethyl–3–(3–dimethylaminopropyl) carbodiimide (EDC) fixative for 60 minutes at room temperature (RT). Sections were washed again, followed by acetylation with triethanolamine and acetic anhydride, to inactivate endogenous alkaline phosphates and peroxidases. After 10 minutes of prehybridization, sections were incubated overnight in 4 pmol of LNA probe diluted in 200 µl hybridization buffer. A hybridization temperature at 20°C below the T_m_ of the experimentally determined miRNA-LNA probe duplex was used. The LNA-probes were synthesized and melting temperatures were experimentally determined in the Tuschl laboratory [45]. After post-hybridization washes, the sections were treated with 3% hydrogen peroxide and washed, before being blocked and incubated with anti-POD-AP for 1 hour at RT (Roche Diagnostics GmbH, Mannheim, Germany). Staining of the sections was done using the TSA Plus Cy3 System (PerkinElmer Life Sciences). Slides for fluorescent staining were mounted with Vectashield® HardSet™ mounting medium with DAPI (Vector Laboratories, Inc., CA, USA).

### Target prediction and pathway analysis

To identify the potentially regulated biological pathways of the Arc-targeting microRNAs they were uploaded into the DIANA-miRpath tool. The microRNAs included were miR-19a, miR-34a, miR-193a and miR-326. The target prediction software used for the pathway analysis was DIANA microT [46]. The pathway analysis performs enrichment analysis of the miRNA input set to all available biological pathways in the Kyoto Encyclopedia of Genes and Genomes (KEGG) [47]. A Pearson's chi-squared test of the input dataset gives the number of genes observed to participate in a given pathway and those expected to occur by chance. The enrichment is represented by a negative natural logarithm of the P-value (−ln P).

## Results

### Multiple miRNAs inhibit Arc 3′UTR reporter expression in HEK cells

**Figure 1:**
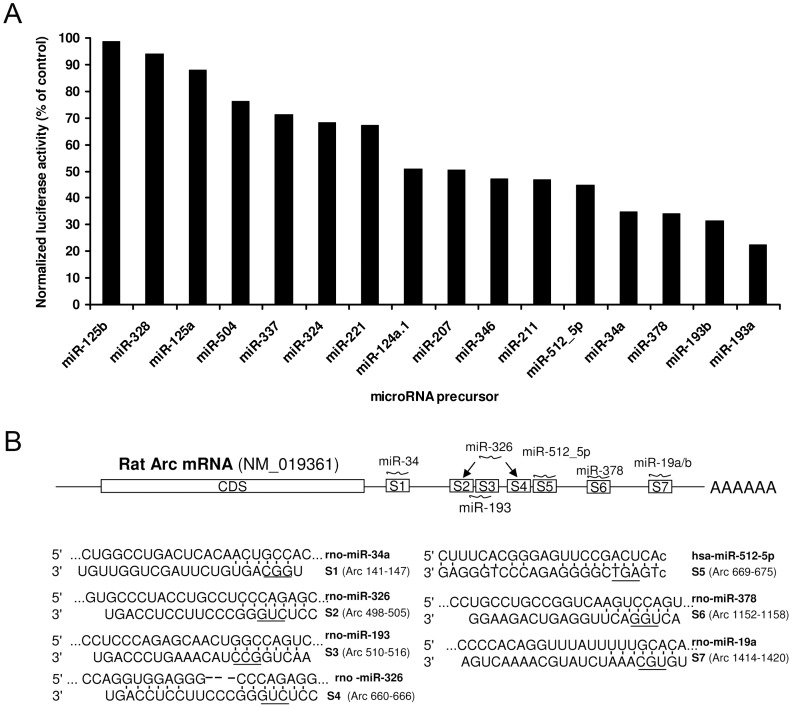
Luciferase reporter screen for microRNAs targeting the Arc 3′UTR. A) Arc 3′UTR luciferase reporter vector and microRNA precursor plasmids were co-transfected in HEK293T cells. After 48 h the luciferase activity was measured and normalized to transfection control (gWIZ, alkaline phosphatase). The luciferase values were further normalized to the average luciferase value obtained after transfecting a panel of microRNAs not predicted to target the rat Arc 3′UTR (rno-miR-370, rno-miR-150, rno-miR-342, rno-miR-30b, rno-miR-105, rno-miR-145 and rno-miR-9). The candidate Arc-targeting microRNAs produced a graded inhibition of Arc 3′UTR luciferase expression ranging from 0 to 70%. B) Schematic representation of the localization of the seven predicted microRNA binding sites in the Arc 3′UTR (NM_019361) that were selected for further studies with site-directed mutagenesis. The numbering refers to the position in the 3′UTR. The nucleotides that were changed by site-directed mutagenesis are underlined in the alignment.

Bioinformatic tools (TargetScan, miRanda and RNA hybrid) based on distinct miRNA target prediction algorithms were used to generate a list of 66 potential miRNA binding sites in the 1626 nucleotide long Arc 3′UTR (NCBI Reference Sequence: NM-019361.1. Sixteen miRNAs with the greatest predicted efficacy of targeting were transfected in HEK 293T cells together with a Gaussia luciferase reporter construct harboring the Arc 3′UTR (Figure 1A). A panel of 7 microRNAs predicted not to bind the Arc 3′UTR was used as a normalization control. The cloned miRNA precursors were confirmed to be overexpressed by qPCR. A dynamic range of regulation was observed for the predicted candidate miRNAs ranging from no effect to a 70% reduction in mean luciferase expression (Figure 1A).

For further validation we selected the four miRNAs (miR-34a, miR-193, miR-378, and miR-512-5p) with strongest inhibitory effect on the Arc 3′UTR. miR-19a and miR-326 were added to this analysis because TargetScan (Release 6.0; November, 2011) shows a vertebrate-conserved binding site for miR-19a and two binding sites for miR-326 in the Arc 3′UTR. A schematic drawing of the Arc mRNA showing the position of the selected miRNA binding sites and alignments between the miRNA sequences and the Arc 3′UTR is shown in Figure 1B.

### Regulation of Arc by miR-34 and miR-326 is attenuated by point mutations and deletion of the microRNA seed region

**Figure 2:**
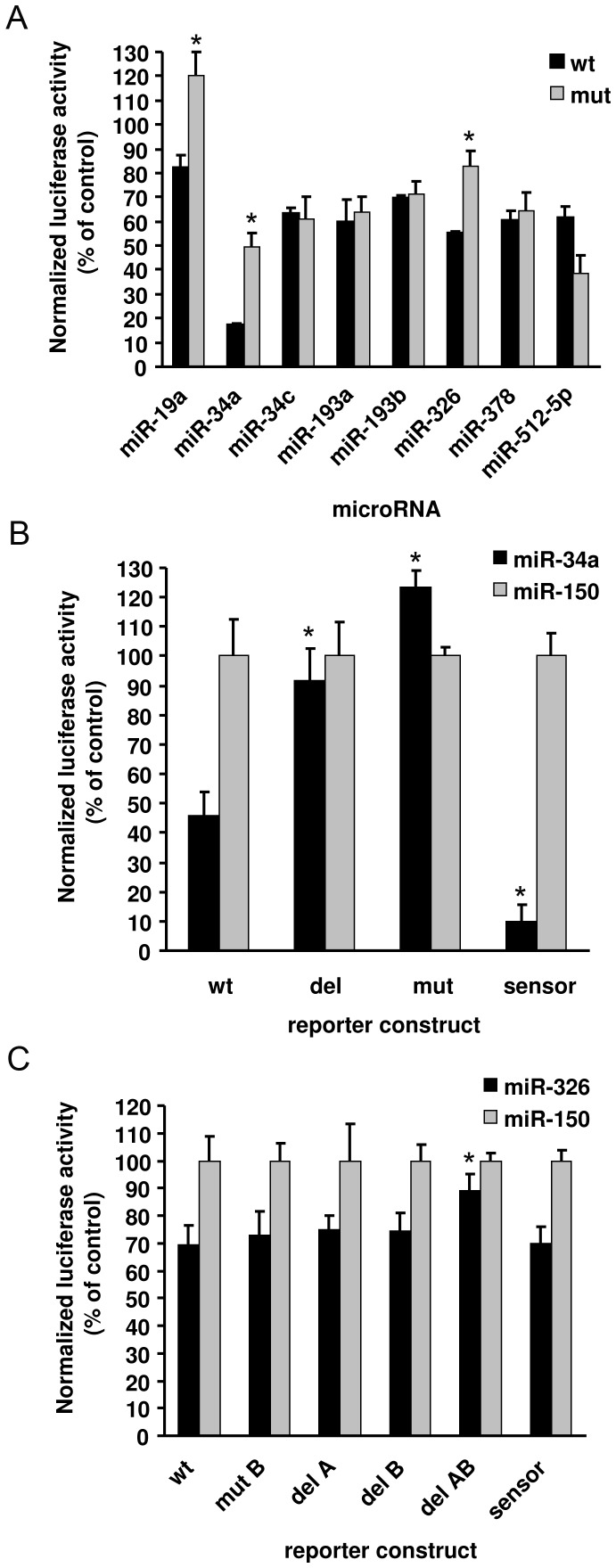
The effects of miR-19a and miR-34a and miR-326 is dependent on intact microRNA binding sites. A) Site-directed mutagenesis was carried out to interfere with 6 different microRNA sites in the Arc 3′UTR. Three nucleotides in the seed-binding region of miR-34, -193, -326, -378 and -512_5p were mutated in the Arc 3′UTR and the whole seed binding region was removed for miR-19. 48 h after co-transfection of HEK293T cells with Arc reporter constructs (wildtype or mutated) and microRNA expression vectors, the medium was harvested for measurement of luciferase and SEAP activity. A significant difference in luciferase expression was observed after substitution mutation or deletion of the miRNA binding sites for miR-19a, miR-34a and miR-326 in response to expression of the respective miRNAs, relative to the wildtype Arc 3′UTR. B) Deletion of the miR-34a site resulted in full recovery of luciferase activity. The positive control comprised of a fully complementary sensor sequence of miR-34a was efficiently inhibited by miR-34a overexpression. C) Whereas mutation of the distal (S4) miR-326 site or deletion of either site individually had only minor effects, the deletion of both sites at the same time gave full recovery of luciferase activity. The sensor construct of miR-326 showed the same effect as deleting both miR-326 binding sites. In panels A, B and C luciferase expression was normalized to the transfection control (gWIZ, alkaline phosphatase) and to miR-150. miR-150 was one of the miRNAs in the initial screen with least effect on the luciferase activity of the reporter vector. Values are means ± SEM (n = 3). * p<0.05, significantly different from wildtype (Student's t-test).

To evaluate the interaction between miRNA and Arc 3′UTR we carried out site-directed mutagenesis (SDM) within the seed-region of the microRNA binding site in the Arc 3′UTR. Triplet substitution mutations were placed within seed nucleotides 2–6 (sites underlined in Figure 1B). These core nucleotides of the seed are necessary for an efficient interaction between the microRNA and the target sequence. Using the wildtype Arc 3′UTR, we independently confirmed inhibition of reporter expression by the candidates selected from Figure 1A and further demonstrated inhibition by miR-19a and miR-326 (Figure 2A). Introducing three point mutations in the binding seed resulted in partial but significant rescue of luciferase expression for miR-34a and miR-326 (Figure 2A). SDM of the proximal miR-326 site (site S2 in Figure 1C) did not affect the luciferase activity (data not shown) whereas mutations directed to the second site resulted in a modest 0.3-fold enhancement in expression relative to wildtype. The impact of miR-34a site mutation was more pronounced, as expression in the mutant reporter was significantly enhanced 2-fold relative to wildtype Arc 3′UTR. The miR-34 family has three members, miR-34a, -34b and -34c, that are predicted to bind the same sites. We therefore compared the effects of ectopic expression of miR-34a and miR-34c on Arc expression. miR-34c reduced wildtype Arc expression to 62% of control levels, whereas miR-34a expression had a much stronger effect, reducing expression to 18% of control. Furthermore, point mutation of the miR-34 binding site rescued inhibition induced by miR-34a, but not miR-34c. Mutation of the binding sites for miR-193, -378 and -512-5p also had no effect on reporter expression elicited by the respective miRNAs.

Introducing nucleotide substitutions in the seed sequence is a way of partially interfering with the binding between microRNA and target. The removal of the whole seed sequence could have a greater impact. Focusing on miR-34a and miR-326, we compared the effect of mutagenesis of three nucleotides in the seed binding region with deletion of the entire seed from the Arc sequence. To confirm functionality of the microRNA overexpression construct we co-transfected a luciferase vector harboring a perfectly complementary binding sequence of the miRNA as a positive control (sensor). For miR-34a, complete recovery of expression was obtained in the substitution and deletion mutants (Figure 2B). Expression of the sensor reporter for miR-34a was effectively inhibited to approximately 10% of control. More moderate effects were obtained for miR-326. Triplet substitution mutation of the distal miR-326 site (S4), deletion of S4, or deletion of S2 failed to enhance expression relative to wildtype control significantly (Figure 2C). However, significant recovery of expression was obtained when both miR-326 binding sites were deleted. Inhibition of the perfectly matched miR-326 sensor was 70% of control, approximately equal to effects obtained with the wildtype Arc 3′UTR reporter. Note, however, that miR-34a gave significantly stronger inhibition of both the wildtype and sensor constructs. For reasons unclear the substitution mutation procedure did not work for miR-19a. However, complete deletion of the miR-19a seed resulted in a significant increase in reporter activity relative to wildtype.

### Arc regulation is enhanced by expression of specific microRNA pairs

**Figure 3:**
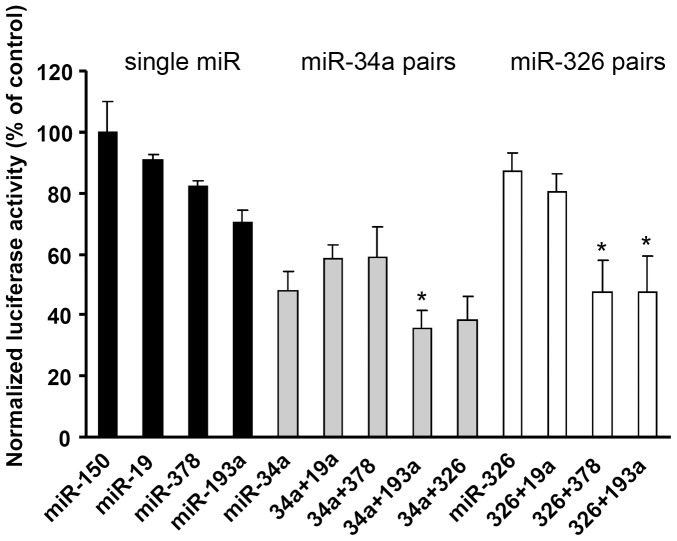
Repression of Arc is enhanced by expression of microRNA pairs. Arc-targeting microRNAs were overexpressed in HEK293T cells alone or in pairs. The following combinations: miR-34a/miR-193a, miR-326/378 and miR-326/193a gave enhanced inhibition of luciferase activity compared to miR-34a and miR-326 alone. Values are means ± SEM (n = 3). *p<0.05, significantly different from miR-34a or miR-326 alone (Student's t-test).

The complexity of the microRNA system and their diverse roles in physiology can partly be explained by their combinatorial effects. Not only can a single microRNA regulate several different target genes, mRNAs often have binding sites for many different microRNAs. We decided to explore possible combinatorial regulation of Arc by miRNAs. Having established the effect of single microRNAs on Arc, we asked if reporter expression is modulated by the expression of specific microRNA pairs (Figure 3). miR-34a and miR-326 were expressed alone and in combination with other Arc-targeting miRNAs. Co-expression of miR-34a with miR-19a, miR-378, or miR-326 did not modulate the inhibition induced by miR-34a expression alone. However, the combination of miR-34a and miR-193a significantly enhanced repression relative to miR-34a alone. Inhibition of the Arc 3′UTR by miR-326 was not affected by co-transfection with miR-19a, yet significantly stronger inhibition was obtained when miR-326 was paired with miR-193a or miR-378. The results from the reporter studies suggest highly specific interactions between miRNA pairs in modulating Arc expression.

### miR-34a, miR-326, and 193a downregulate Arc protein in hippocampal neurons

**Figure 4:**
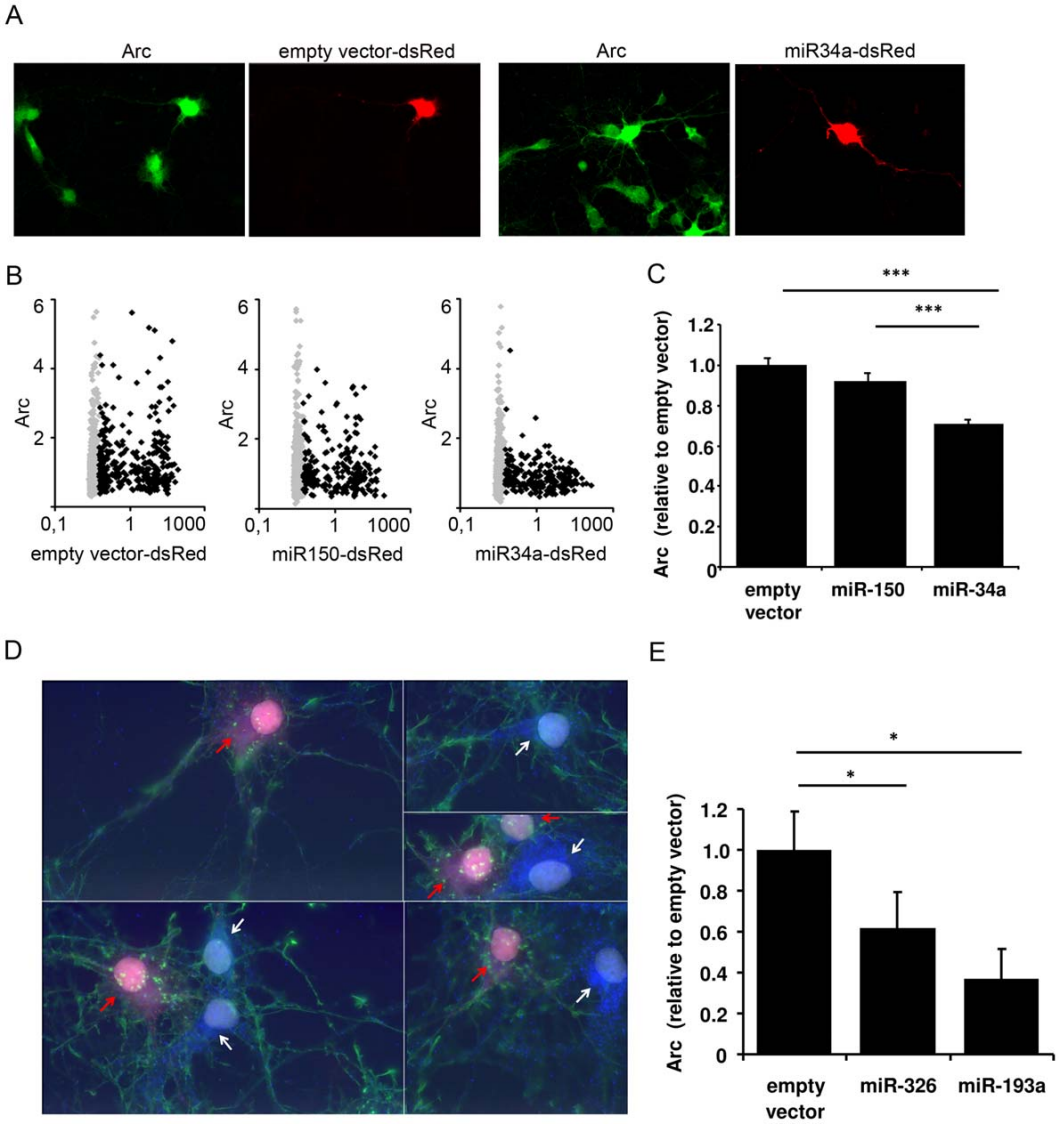
miR-34a, miR-326 and miR-193a downregulate Arc protein expression in cultured hippocampal neurons. Cultured hippocampal neurons were transfected with either empty vector-DsRed, miR150-DsRed, miR34a-DsRed, miR326-DsRed or miR193a-DsRed. Neurons were treated with BDNF for four hours and Arc protein expression was assessed by light microscopy. A) representative images of cells transfected with empty vector-DsRed and miR34a-DsRed, respectively. Note that Arc levels are very diverse within one sample. The fields of view were chosen to contain at least one DsRed-expressing neuron per image, and approximately 60 images from at least two different coverslips were taken per condition per experiment. During image acquisition and subsequent data analysis, the experimenter was blinded to the treatment group of the cells. B) scatter plots of Arc vs. DsRed levels per cell. Data are normalized to the average value of non-transfected cells. Gray diamonds = non-transfected cells; black diamonds = transfected cells. n = 3996 cells from 3 experiments. C) bar graph comparing the average Arc level in transfected cells. Values are normalized to the average Arc level in the non-transfected cells. A significant downregulation of Arc protein was seen in miR-34a transfected cells. Significance was tested by independent t-tests, p<0.001. Error bars = SEM. n = 763 cells from 3 experiments. D) representative images of cells transfected with miR-326-DsRed (cells labelled with DsRed and red arrows) and non-transfected cells (white arrows). Immunostaining of Arc protein is visualized in blue. Note that Arc levels are very diverse within one sample. Phalloidin labelling in green was used to visualize the individual cells. E) bar graph comparing the average Arc level in transfected cells. Values are normalized to the average Arc level in the non-transfected cells. A significant downregulation of Arc protein was seen in miR-193a and miR-326 transfected cells. Significance was tested by univariate ANOVA and post hoc tests, p<0.05. Error bars = SEM. n = 339 cells from 3 experiments.

Next we asked if overexpression of these miRNAs regulates endogenous Arc expression in neurons. For this purpose E18 primary hippocampal neuronal cultures were transfected with microRNA expression plasmids, yielding sparsely transfected neurons suitable for analysis by fluorescence microscopy. DIV7 cultures were transfected with plasmids expressing empty vector, the control microRNA miR-150, or an Arc-targeting microRNA. We chose to study miR-34a and miR-326 as strong candidates from the point mutation analysis and miR-193a because of its synergistic effects. A red fluorescent label, DsRed, was present on the plasmids to mark the transfected cells. Arc expression was induced by a four-hour treatment with BDNF prior to paraformaldehyde fixation and imaging of the neurons. While there was considerable cell-to-cell variation in immunofluorescence for Arc within treatment groups (Figure 4A and D), automated cell segmentation of a high sample number demonstrated significant modulation of endogenous Arc protein expression consistent with effects obtained in heterologous cells. The levels of Arc protein was plotted against the level of DsRed expressed after transfection (Figure 4B). Mean Arc levels were comparable between cells transfected with empty vector and miR-150 controls, but were significantly reduced in neurons transfected with miR-34a, miR-326, or miR-193a (Figure 4C and E). Inhibition ranged between 30 and 60%.

### Peptide-nucleic acid miRNA inhibitors enhance endogenous Arc mRNA expression

**Figure 5:**
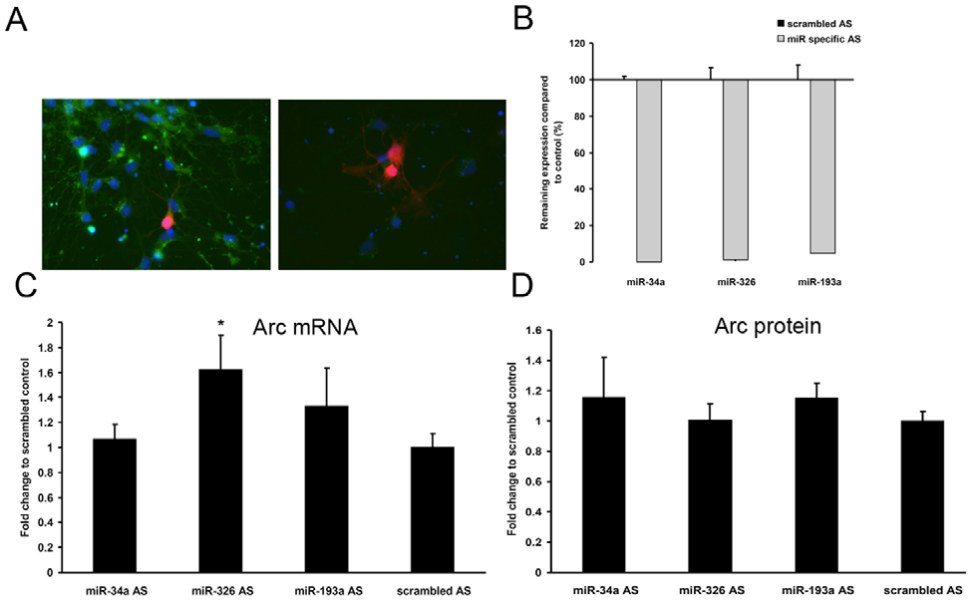
Inhibition of endogenous Arc-targeting miRNAs in hippocampal neurons. PNA-conjugated antisense oligonucleotides were used to block endogenous microRNAs. A) The uptake of a fluorescent control PNA oligo is efficient in hippocampal neurons (DIV8) 24 hours after transfection. Green fluorescence = PNA. DsRed was cotransfected to compare the level of PNA transfection with that of plasmid. B) RNA enriched for small RNAs was isolated 48 h after transfection of antisense and scrambled control PNA and the level of unbound microRNAs was assayed by real-time PCR. 1% of miR-326, 0.7% of miR-34a and 4.7% of miR-193a remained unblocked after specific PNA transfection compared to control. Y1 was used for normalization. Values are means of n = 3–4 ± SEM C) RNA was isolated 48 h after transfection of microRNA inhibitors and used for Arc mRNA Q-PCR. Arc mRNA is significantly higher after transfection of miR-326 PNA-AS compared to scrambled (p = 0.04). Significance was tested by independent t-tests. Data is normalized to polyubiquitin and cyclophilin. Values are means of n = 5 ± SEM. D) Proteins were harvested 48 h after PNA transfection. Bar graphs representing densitometry measurements from protein western blot analysis. The Arc expression was normalized to the expression of GAPDH. Values are means of n = 7 ± SEM.

Next we sought to inhibit the endogenous miRNAs by overexpressing antisense oligonucleotides in hippocampal neurons while measuring the effect on Arc mRNA and protein expression by qPCR and western blot. Such regulation may be difficult to detect in lipofectamine transfected neurons with transfection rates typically below 5%. For that reason we combined lipofectamine transfection with peptide nucleic acid (PNA™) antisense oligonucleotides (PNA-AS) to inhibit endogenous miRNAs. In PNAs, cellular uptake is facilitated by conjugation of a cell-penetrating peptide (CPP) to the antisense oligo. Moreover, replacement of the phosphate ribose ring of the antisense oligo with a polyamide backbone of N-(2-aminoethyl) glycine confers greater binding affinity and stability [48]. The transfection efficiency of a fluorescein-labeled PNA oligo in hippocampal neurons was approximately 80% at 24 hours after transfection (Figure 5A). Unstimulated hippocampal neurons were transfected at DIV8 with PNA-AS complementary to miR-34a, miR-326 and miR-193a and cells were harvested 48 hours later for qPCR or western blot. Arc mRNA was significantly increased after treatment with miR-326, but not miR-34a, PNA-AS (Figure 5C). Transfection of miR-193a AS resulted in a small but insignificant increase in Arc mRNA. Surprisingly, no effects were detected at the protein level (Figure 5D). PNA-AS bound to target microRNAs should prevent detection of the miRNA by qPCR. We confirmed that PNA-AS transfection almost completely eliminated PCR-detectable miR-326, miR-34a and miR-193 (Figure 5B). The corresponding miRs were observed at considerably higher Ct values after transfecting miR specific PNAs compared to control (AS-miR34a Ct = 36; scrambled Ct = 27, AS-miR-326 C = 40; scrambled CT = 30, AS-miR-193 CT>40; scrambled CT = 35).

### Developmental regulation of Arc-targeting miRNAs in hippocampal neurons *in vitro*

**Figure 6:**
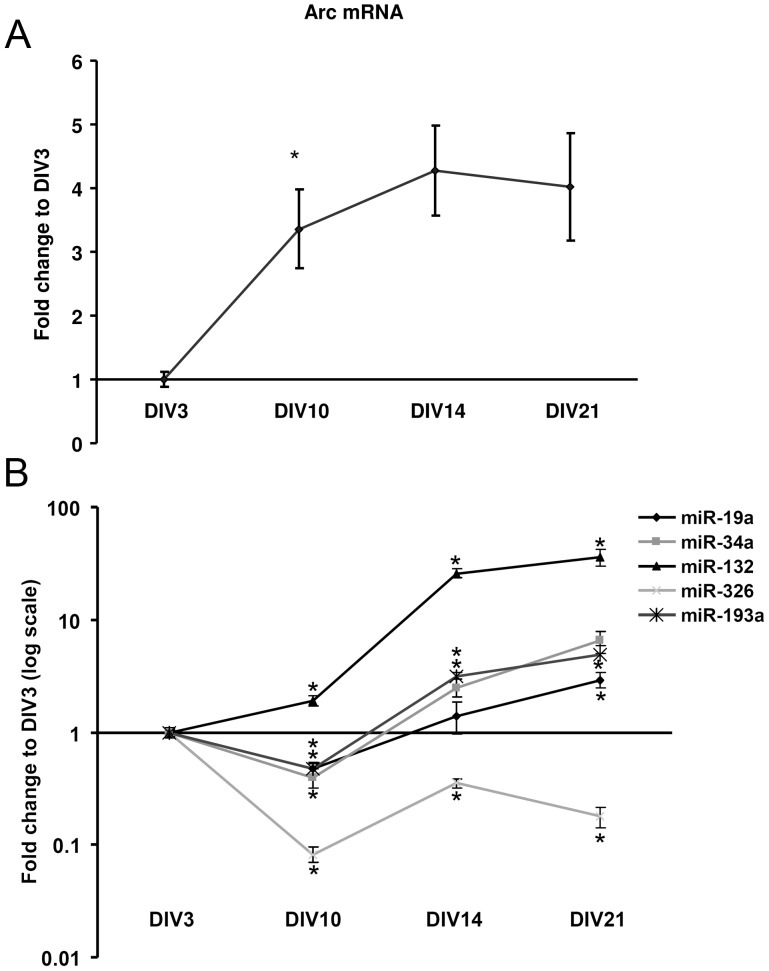
Developmental regulation of Arc-targeting microRNAs. Arc mRNA expression and mature microRNA levels in hippocampal neurons from E18 rat embryos at different developmental stages (days *in vitro*, DIV). A) Quantitative relative real-time PCR of Arc mRNA. Arc mRNA increased at early time points and reached a plateau around DIV14. The relative values are expressed as fold change to DIV3 and normalized to the geometric mean of the reference genes HRPT and cyclophilin. B) Quantitative relative real-time PCR of miR-19a, miR-34a, miR-326, miR-193a and miR-132. The relative values are expressed as fold change to DIV3 and normalized to the reference genes snoRNA and Y1. At early stages of differentiation there was an inverse relation between the expression of Arc mRNA and miR-34a, -19a, -326 and -193a expression. miR-132 expression increases with neuronal differentiation. Note that the y-axis scale is in log format in B. In both A and B significance was tested by independent t-tests, p<0.05. Values are means of n = 4 ± SEM. * significantly different from the preceding time point.

In the next series of experiments, we sought to gain insight into the regulation of the Arc-targeting microRNA *in vitro* and *in vivo*. We first examined the developmental regulation of miRNA expression in hippocampal neurons *in vitro*. Levels of Arc mRNA and mature miRNAs were determined by qPCR in E18 hippocampal neuronal cultures at DIV 3, 10, 14 and 21. Arc mRNA levels elevated at day 10, 14, and 21, relative to day 3 (Figure 6A). Next we looked at the levels of three Arc-targeting miRNAs over the same time frame. Dramatic changes in mature miR-19a, miR-34a, miR-326 and miR-193a were observed during development, with maximum changes of more than 100-fold. Note that miRNA expression is reported with a log scaled y-axis. At DIV10, all three miRNAs exhibited significantly decreased expression relative to DIV3 (Figure 6B). After day 10, the expression of the miRNAs increased, though levels of miR-326 remained significantly decreased relative to DIV3. Thus, at early stages of differentiation (DIV3-DIV10) there is an inverse relationship between expression of Arc-targeting miRNAs and levels of Arc mRNA. As a positive control we also examined miR-132, which is known to be developmentally upregulated in hippocampal neurons [49], [50]. We show that, in contrast with the Arc-targeting miRNAs, miR-132 expression progressively increases during the first three weeks of *in vitro* development (Figure 6B).

### BDNF regulates expression of Arc mRNA, but not of Arc-targeting miRNAs, in hippocampal neurons

**Figure 7:**
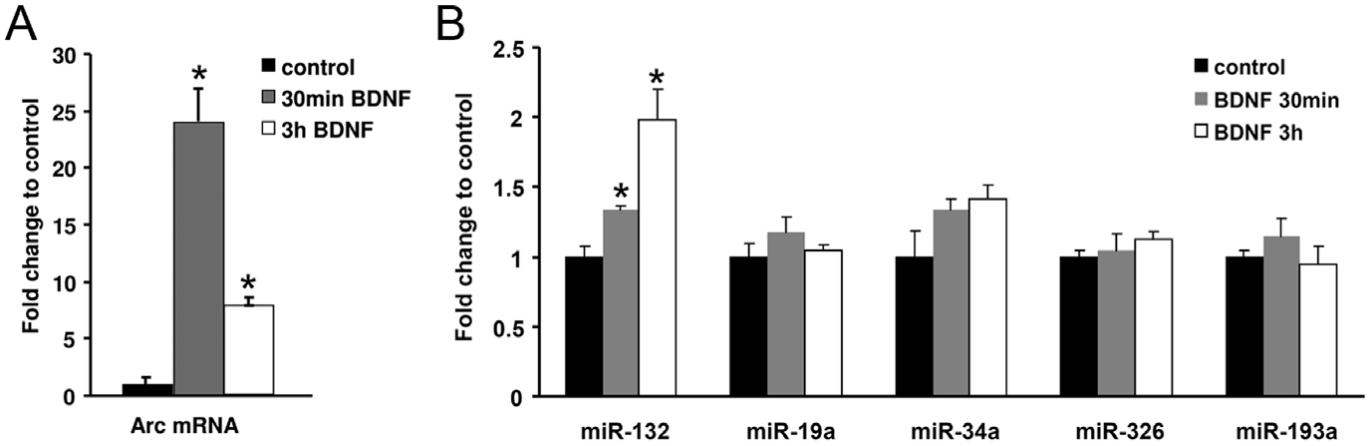
BDNF does not alter levels of mature Arc-targeting miRNAs. The effect of BDNF stimulation (for 30 minutes or 3 hours) on microRNA and Arc expression was studied at DIV8. A) Quantitative relative real-time PCR of Arc mRNA. The relative values are expressed as fold change to untreated control cells and normalized to the reference genes HRPT, polyubiquitin and cyclophilin. Arc mRNA levels are significantly increased at 30 minutes and 2 hours of BDNF treatment. B) Quantitative relative real-time PCR of miR- miR-133, 19a, miR-34a, miR-326 and miR-193a. The relative values are expressed as fold change to untreated control cells and normalized to the reference genes snoRNA and Y1. The expression of Arc-targeting miRNAs did not change in response to BDNF. Values are means of n = 4 ± SEM. In both A and B significance was tested by independent t-tests, p<0.05. * significantly different from control.

Brain-derived neurotrophic factor (BDNF) can induce the expression of plasticity-related genes such as Arc and miR-132 in embryonic hippocampal neurons [39], [49]–[51]. We therefore investigated the effect of BDNF treatment of hippocampal neurons on expression of Arc-targeting miRNAs, using Arc mRNA and miR-132 as positive controls. DIV8 neurons were treated with BDNF for 30 minutes or 3 hours. Arc mRNA was elevated 24-fold above non-treated control neurons after 30 minutes of BDNF treatment, declining to a 7-fold increase at 3 hours. Using the same RNA preparations from whole lysates, we confirmed significant upregulation of miR-132 at the 30 minutes (1.4 fold) and 3 hours (2-fold) time points (Figure 7B). In contrast, Arc-regulating miR-34a, miR-326, miR-19 and miR-193a were not significantly regulated, although there was trend for miR-34a upregulation at 30 minutes post-BDNF (p = 0.07).

### LTP induction in the dentate gyrus of adult anesthetized rats upregulates Arc mRNA without regulating corresponding miRNAs

**Figure 8:**
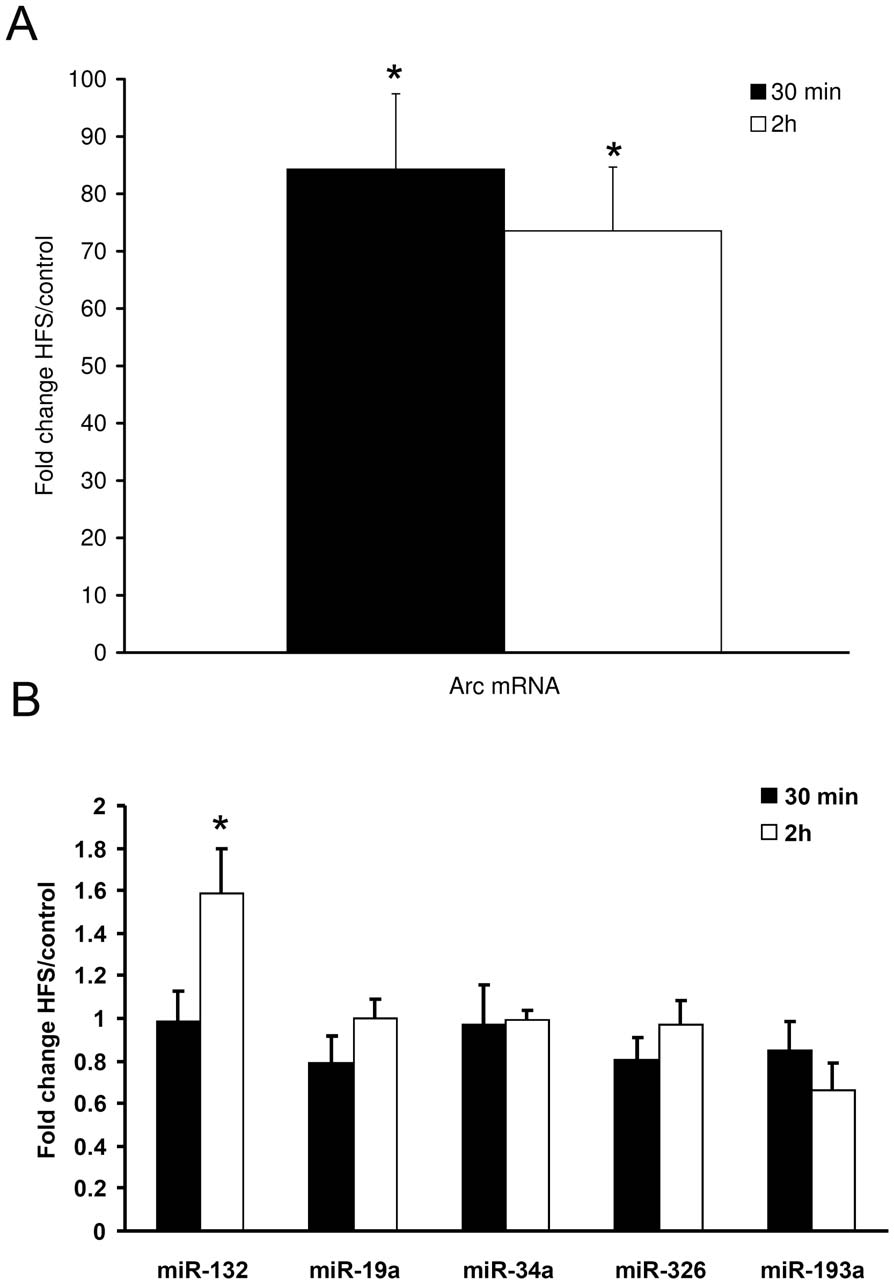
LTP-inducing stimulation does not alter the levels of mature Arc-targeting miRNAs. The effect of LTP induction in the dentate gyrus on miRNA and Arc expression was studied by real-time PCR analysis of dentate gyrus samples obtained 30 min and 3 hours after high-frequency stimulation (HFS) of the perforant path. A) Real-time PCR of Arc mRNA. Bar graphs indicate fold change in the HFS-treated dentate gyrus relative to the control, contralateral dentate gyrus. Arc mRNA is induced 80-fold after HFS. Values are means of n = 2 ± SEM. The data was normalized to the reference genes HRPT, polyubiquitin and cyclophilin. B) Quantitative relative real-time PCR of miR-19a, miR-34a, miR-326, miR-193a and miR-132. miR-132 was significantly elevated at 2 h post-HFS. Bar graphs indicate fold change values comparing treated dentate gyrus relative to control. Values are means of n = 5 ± SEM. Data has been normalized to the reference genes Y1. Significance was tested by independent t-tests. * p<0.05 and significantly different from control.

We then turned to examine microRNA regulation in the intact adult brain. We asked whether Arc-targeting miRNAs are regulated *in vivo* by synaptic stimulation, using LTP in the dentate gyrus of anesthetized rats as a model system. Brief bursts of high-frequency stimulation (HFS) applied to the medial perforant path generated a lasting increase in the slope of the field excitatory postsynaptic potential (fEPSP). Previous work has shown that Arc mRNA is strongly induced and transported to dendrites following HFS, while Arc translation is necessary for LTP consolidation [7], [42], [43], [52]. LTP in the dentate gyrus is also associated with upregulation of mature miR-132 at two hours post-HFS [23]. In the current study, qPCR analysis performed on whole dentate gyrus lysates confirmed upregulation of Arc mRNA at 30 minutes and 2 hours post-HFS and upregulation of miR-132 at 2 hours (Figure 8A and 8B). In contrast, miR-19a, miR-34a, miR-326 and miR-193a were not significantly regulated. We conclude that robust upregulation of Arc mRNA levels is not associated with detectable changes in the expression of several mature miRNAs capable of regulating Arc.

### Arc-targeting miRNAs exhibit a somatodendritic distribution and synaptoneurosomal localization

**Figure 9:**
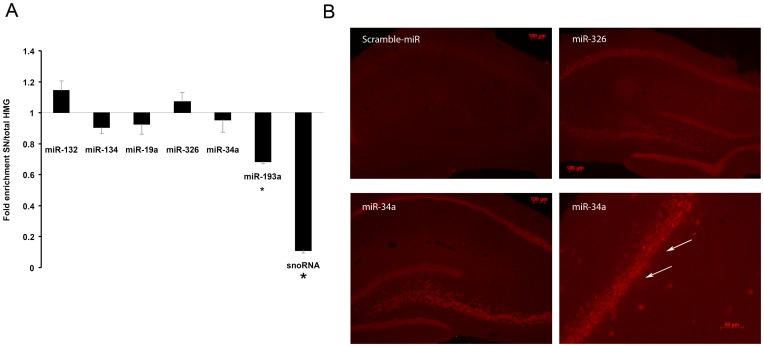
Synaptic expression of Arc-targeting microRNAs. A) Synaptoneurosomes (SNs) were prepared from normal untreated dentate gyrus and semi- quantitative relative real-time PCR was used to assess the synaptic localization of Arc-targeting miRNAs. Selected miRNAs were assayed in total homogenate and in the synaptoneurosome fraction. miR-124a was equally expressed in both preparations and used to normalize the data. Small nucleolar RNA (snoRNA) was highly depleted from the SN fraction and served as a quality control of the preparation. In contrast, the Arc-associated miRNAs and a known dendritic miRNA, miR-132, showed similar SN/homogenate expression ratios. The relative values are expressed as the fold enrichment between synaptoneurosomes and total homogenate. n = 6 Significance was tested by independent t-tests, p<0.05. *Significantly different from total homogenate. Error bars = SEM. B) miRNA *in situ* hybridization was performed on coronal hippocampal sections using LNA probes for miR-326, miR-34a, and scrambled control. miR-326 and miR-34a showed specific staining in the cell body layers of the dentate gyrus and cornu ammonis (CA) regions compared to scramble control. For miR-34a, the staining was observed approximately 30 µm into the apical dendrites of CA1 pyramidal cells. Lower right panel shows CA1 region with examples of proximal dendritic staining marked by white arrows.

Arc mRNA is transported to dendrites where it can be locally translated. If miRNAs directly regulate local Arc translation they should be present in dendrites. We therefore examined the localization of Arc-targeting miRNA using qPCR analysis of the dentate gyrus synaptoneurosome fraction and miRNA *in situ* hybridization of brain sections. Synaptoneurosomes (SNs) were derived by subcellullar fractionation of whole dentate gyrus lysates. The SN fraction is enriched in pinched-off, resealed dendritic spines attached to presynaptic boutons. Semiquantitive RT-PCR was used to compare miRNA levels between the SN and total homogenate. miR-124 was used as normalization control because previous studies showed that miR-124 is neuron-specific and stably expressed in SNs and homogenates obtained from adult mouse forebrain [53]. We similarly observed equivalent expression of miR-124 in SNs and lysate samples. To further validate the preparation we measured levels of small nucleolar RNA (snoRNA), a strictly nuclear RNA present in neurons and glia. As shown in Figure 9A, the Arc-targeting microRNAs (miR-19a, -32a, -326, -193a), exhibited SN/homogenate expression ratios near 1 and were all significantly different from snoRNA with an expression ratio of 0.15. The expression of Arc-targeting miRNAs was not significantly different from that of the previously described dendritic miRNAs, miR-132 and miR-134.

miRNA *in situ* hybridization was performed in coronal sections of perfusion-fixed brains using specific locked-nucleic acid probes (Figure 9B). Prominent miR-34a and miR-326 expression was observed in the cell body layers of the dentate gyrus and hippocampus proper. Dentate granule cells and hippocampal pyramidal cells showed clear cytoplasmic, punctate staining. miR-34a staining was also apparent in the proximal apical dendrites of pyramidal cells, but not in dendrites of granule cells. There was also no detectable dendritic staining for miR-326 (Fig. 9B). Taken together, the results suggest that under basal (non-stimulated) conditions the levels of miR-34a and miR-326 in distal dendrites is much lower than in the cell body. This smaller pool of miRNAs is detectable by qPCR in synaptoneurosomes but is probably below the detection threshold for fluorescence *in situ* hybridization.

## Discussion

The present study identifies microRNA regulators of Arc and elucidates basic features of their activity-dependent expression and subcellular localization. There are four main conclusions from this work. 1) Arc expression is regulated by multiple miRNAs. This was shown by ectopic miRNA expression in HEK cells and hippocampal neurons and by inhibition of the endogenous miRNAs in neurons. In addition, additive effects were obtained by ectopic expression of specific miRNA pairs in HEK cells. 2) During *in vitro* neuronal development there is an inverse relationship between Arc mRNA expression and expression of Arc-targeting miRNAs. Thus, at DIV10, expression of miR-19a, miR-34a, miR-326 and miR-193a were decreased while Arc mRNA was elevated. 3) Activity-dependent expression of Arc is not accompanied by changes in steady state levels of mature, Arc-targeting miRNAs. This conclusion is based on measurements of miRNA levels in lysate samples from hippocampal neuronal cultures treated with BDNF and from dentate gyrus after LTP induction *in vivo*. Notably, both treatment paradigms induced the BDNF-regulated miRNA, miR-132, in addition to Arc mRNA. 4) Finally, Arc-targeting miRNAs are synaptically expressed. *In situ* hybridization confirms miRNA expression in adult dentate granule cells and hippocampal pyramidal cells, while qPCR analysis shows enhanced expression of miR-19a, miR-34a, and miR-326 in synaptoneurosomes relative to cell body restricted, small nucleolar RNA.

Using an Arc 3′UTR luciferase reporter in HEK cells we show graded regulation of 16 miRNAs with predicted binding sites in Arc. Mutations in the seed region of three miRNAs (miR-34a, miR-326, and miR-19a) partially or fully rescue reporter expression. In hippocampal neurons, miRNA overexpression inhibits endogenous Arc protein expression in response to BDNF, while inhibition of miR-326 facilitates Arc mRNA expression. Collating results from mutation studies in HEK cells with effects of miRNA manipulation in hippocampal neurons, we provide evidence that miR-19a, miR-34a, miR-193a, and miR-326 are capable of modulating Arc. Our analysis focused on a subset of miRNAs that most effectively inhibit reporter expression or which have a vertebrate-conserved binding site in Arc. It is certainly possible that other microRNAs, though less effective in the reporter assay, also modulate Arc.

Arc is a highly regulated gene system essential for long-term synaptic plasticity, memory, and other adaptive changes in the nervous system. Arc mRNA is induced as an immediate early gene and transported to dendrites for local translation and degradation, and Arc protein synthesis is necessary for consolidation of LTP, long-term depression (LTD), and homeostatic plasticity. For these reasons, Arc is attractive as a model for elucidating how miRNAs interact with activity-induced mRNAs in the regulation of local protein synthesis and specific functional outcomes like LTP and LTD. Questions abound regarding the logic of Arc-targeting miRNAs: 1) Are these miRNAs co-induced with Arc mRNA? 2) Are the mature miRNAs deposited onto nascent Arc mRNA in the cell body or after transport of RNA into dendrites? 3) Are miRNAs primarily used to silence Arc or as platforms for activity-dependent derepression?

As recruitment of the miRISC could potentially destabilize Arc mRNA and repress translation, it is relevant to consider current knowledge of Arc mRNA stability and translational control. Previous work has identified Arc mRNA as a physiological target for nonsense-mediated RNA decay (NMD) [17]. The Arc 3′UTR has two introns and splicing of these introns results in the deposition of the exon-junction complex near the stop codon and recruitment of the NMD machinery during the first round of translation. This form of translation-dependent degradation may explain the rapid turnover of Arc mRNA and the tight coupling between Arc transcription and protein expression during LTP consolidation in the dentate gyrus in vivo [7], [15], [43]. Looking at translation, *in vitro* studies show that specific Arc translation is regulated at least in part by the RNA-binding protein, fragile-X mental retardation protein (FMRP), which binds to the Arc 3′UTR and to cytoplasmic FMRP-interacting protein (CYFIP) [54]. By binding to eIF4E on the 5′cap, CYFIP prevents translation of FMRP bound mRNAs. This repression is relieved by BDNF and metabotropic glutamate receptor signaling which dislodge CYFIP/FMRP from the cap structure [54]. During in vivo LTP, ERK signaling to MAP-kinase interacting kinase (MNK) controls LTP consolidation, initiation complex formation, and Arc synthesis [43]. Moreover, MNK triggers the release of CYFIP/FMRP from eIF4E (Panja and Bramham, unpublished). Arc also has a cis-acting A2 response-element (A2RE) in the coding region that is specifically recognized by the heterogenous ribonucleoproteins hnRNP A2 and hnRNP A/B and which functions in the dendritic transport of Arc mRNA [55], [56].

Functional interactions between miRNAs and RNA-binding proteins have been shown for FMRP [57] and HuR, an AU-rich element binding protein [58], [59]. Theoretically, the additive effects of specific miRNA pairs in regulating Arc could be related to the position of the miRNA binding sites relative to each other and to binding sites of RNA-binding proteins. However, there was no obvious positional relationship that might explain our observations. miR-193a enhanced repression by both miR-34a and miR-326. The seed binding site of miR-193a is close (5 nts) to the first miR-326 site but 360 nts distant from miR-34a. miR-378 and miR-34a also have a synergistic effect yet bind to quite widely separated (1000 nt) sites. FMRP is of particular interest given work on miRNA regulation of PSD95 translation in neurons, but the FMRP recognition motif on the Arc 3′UTR has yet to be determined. The A2 response element in the coding region cannot be a factor in our experiments on the Arc 3′UTR reporter, although the potential for miRNA regulation within the coding region merits consideration.

Regarding the question of Arc/miRNA co-induction, the present work demonstrates that Arc is robustly upregulated without a concomitant change in the expression of the mature miRNAs. This stands in contrast to miR-132 which is transcribed in a synaptic activity-dependent manner and rapidly functions to repress protein expression of synaptic proteins such as p250GAP [60]. In terms of regulation of mRNA stability versus translation, we show that inhibition of miR-326 in unstimulated neurons enhances Arc mRNA expression without affecting protein, indicating that miR-326 may specifically function to destabilize Arc mRNA. The lack of effect of PNA-AS on Arc protein is surprising, but may be related to the observed low levels of endogenous miRNA expression at DIV10. Ectopic expression of miR-193a, miR-326, and miR-34a all enhanced BDNF-evoked Arc protein expression.

Research on miRNA metabolism in the nervous system has focused primarily on biogenesis. However, the stability of mature miRNA also appears to be highly regulated. While many miRNAs are stable for long periods (weeks) in many cell types, recent work suggests that some neuronal microRNAs turnover on the order of minutes [23], [25], [27], [61]. For example, mature miR-219 is downregulated 2 hours following NMDA receptor-dependent LTP induction without any change in primary and precursor miR-219 [23]. The present work shows that Arc-targeting miRNAs are constitutively expressed in the synaptoneurosome fraction. The fact that these miRNAs are not co-induced with Arc mRNA in whole dentate gyrus lysates favors the notion that Arc repression/derepression is mediated by pre-existing mature miRNA, or by local synaptic changes in miRNA metabolism.

miRNAs are often thought to act in a combinatorial manner on multiple targets to sculpt protein expression that determines specific cellular phenotypes or functions.

DIANA pathway analysis was done to predict biological processes regulated by the five Arc-related miRNAs. Among the top 10 process predicted were long-term potentiation, regulation of the actin cytoskeleton, axon guidance, and ERK signaling (Supp Figure S1). Interestingly miR-19, miR-34 and miR-326 are all dysregulated in multiple sclerosis patients [62]. While little is currently known about mir-193a and miR-19a, new studies have shed light on miR-34 and miR-326 function in the nervous system.

miR-34 family microRNAs (miR-34a, -34b, -34c) share a common seed sequence and therefore share many of the same targets in many cell types. For instance, all three miR-34s cooperate in inhibition of somatic cell reprogramming [63]. miR-34a and 34b/c are suppressive in many tumor types and they inhibit the epithelial-mesenchymal-transition [64]–[66]. Targets of known or potential relevance in neuronal function and plasticity include Notch1, histone deacetylase sirtuin1 (SIRT1), and growth factor signal transduction [67]. However, the miR-34 family genes are differentially expressed and regulated. miR-34b and -34c are co-transcribed from the same cluster distinct from the miR-34a gene. In brain, miR-34a is developmentally upregulated through embryonic and post-natal maturation under control of the p53 family transcription factor, TAp73 [68], [69]. miR-34a negatively regulates dendritic branching of immature cortical neurons *in vitro* and downregulates expression of synaptotagmin and syntaxin. miR-34a and miR-34c are also coexpressed following acute restraint stress and chronic social defeat stress in mice. Virally-mediated expression of miR-34c in the central nucleus of the amygdala has anxiolytic effects, possibly through regulation of corticotropin releasing factor [70]. Zhou et al showed that the mood stabilizing drugs lithium and valproate downregulate miR-34a, which in turn regulates expression of metabotropic glutamate receptor 7 in hippocampal neurons [71]. Recent work elegantly revealed a role for miR-34c in contextual fear conditioning, and identified SIRT1 as a decisive target for this regulation [30]. The authors point out that miR-34c may have other targets, referring specifically to c-MYC [30], [72]. Interestingly, Arc synthesis is also required for contextual fear conditioning [73], and the present work identifies Arc as another potentially important target for miR-34a. In the present luciferase reporter analysis, overexpression of mir-34a and -34c both inhibited Arc, but only the 34a-mediated inhibition was reversed by substitution mutation of the seed region, suggesting a preferential interaction of miR-34a with Arc.

miR-326 is expressed in neurons and works in a feedback loop with Notch during development of the nervous system [74]. In the adult brain, Notch is necessary for LTP and LTD, and proteolytic activation of Notch in dendrites is dependent on Arc [75]. Thus, miR-34a and miR-326 have Arc and Notch as common targets. TargetScan further predicts a binding site for miR-326 in two dendritically expressed RNAs with roles in synaptic plasticity, protein kinase C-zeta and tissue plasminogen activator.

Future goals are to delineate the function of specific miRNA-Arc interactions, and how this relates to the transcription, processing, and decay of the miRNAs.

## Supporting Information

Figure S1 **Pathway analysis of Arc-targeting microRNAs.** Pathway analysis of Arc-targeting microRNAs was done on DIANA mirPath using DIANA microT (Beta version) as the target prediction tool. Histograms show different predicted pathways potentially regulated by combined expression of the miRNAs the score obtained is represented as −ln(P-value). Pathways with top ten scores have been plotted against −ln(P-value).
(TIF)

Table S1 **Primer sequences and accession numbers for genes analyzed.**
(DOC)
