# Supplementary figures and images for: MicroRNA Regulation of the Synaptic Plasticity-Related Gene Arc
Source: PLoS One. 2012 Jul 26;7(7):e41688. doi: 10.1371/journal.pone.0041688 (PMC3406043; doi:10.1371/journal.pone.0041688)

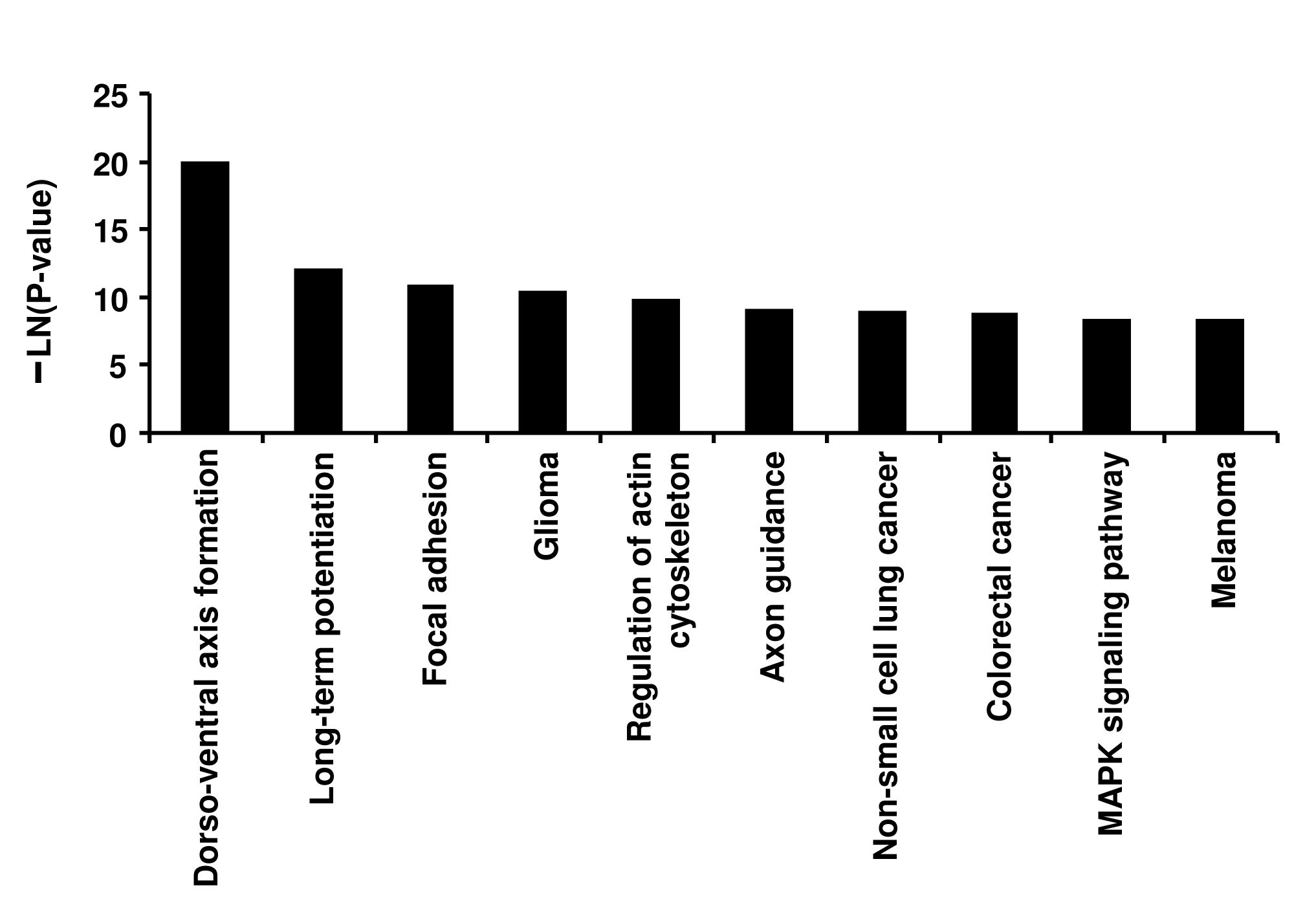

Supplement: Figure S1 — Pathway analysis of Arc-targeting microRNAs. Pathway analysis of Arc-targeting microRNAs was done on DIANA mirPath using DIANA microT (Beta version) as the target prediction tool. Histograms show different predicted pathways potentially regulated by combined expression of the miRNAs the score obtained is represented as −ln(P-value). Pathways with top ten scores have been plotted against −ln(P-value). (TIF) [file pone.0041688.s001.tif]
