# Supplementary material for: MicroRNA Regulation of the Synaptic Plasticity-Related Gene Arc
Source: PLoS One. 2012 Jul 26;7(7):e41688. doi: 10.1371/journal.pone.0041688 (PMC3406043; doi:10.1371/journal.pone.0041688)
Supplement: Table S1 — Primer sequences and accession numbers for genes analyzed. (DOC) [file pone.0041688.s002.doc]

**Supplemental Table S1. Primer sequences and accession numbers for genes analyzed with PCR**

| **Gene** | **Primers** | **Acc number** |
| --- | --- | --- |
| Activity-regulated cytoskeleton-associated protein | FW: CCCAGTCTGTGGCTTTTGTCA  BW: GTGTCAGCCCCAGCTCAATC | NM019361 |
| Polyubiqutine | FW: GGCAAGACCATCACCCTAGA  BW: GCAGGGTTGACTCTTTCTGG | BC070919 |
| Cyclopholine | FW: AGCACTGGGGAGAAAGGATT  BW: GATGCCAGGACCTGTATGCT | BC059141 |
| Hypoxanthine phosphoribosyltransferase | FW: GCAGACTTTGCTTTCCTTGG  BW: TCCACTTTCGCTGATGACAC | NM012583 |
